# A Dominant Probiotic from the Traditional Chinese Fermented Food Douzhier Exhibits Anti-Influenza Activity

**DOI:** 10.3390/nu18183089

**Published:** 2026-09-21

**Authors:** Xiao Zhang, Yuewen Yang, Siqin He, Yuanming Huang, Zhihong Ren, Liqiong Song

**Affiliations:** 1 National Key Laboratory of Intelligent Tracking and Forecasting for Infectious Diseases, National Institute for Communicable Disease Control and Prevention, Chinese Center for Disease Control and Prevention & Chinese Academy of Preventive Medicine, Beijing 102206, China; zx19909440050@163.com (X.Z.); yangzishun123@163.com (Y.Y.); huangyuanming@icdc.cn (Y.H.); 2 School of Public Health, Gansu University of Chinese Medicine, Lanzhou 730000, China; 3 Hunan Prevention and Treatment Institute for Occupational Diseases, Affiliated Prevention and Treatment Institute for Occupational Diseases of University of South China, Changsha 410007, China; hesiqin97@foxmail.com

**Keywords:** Douzhier, *Lactococcus cremoris* D2022, probiotic, influenza A virus, gut microbial metabolites

## Abstract

**Background:** Douzhier, a traditional Chinese fermented food with a history spanning over 1000 years, harbors a complex community of beneficial microbiota. In this study, we isolated *Lactococcus lactis* subsp. *cremoris* (designated *L. cremoris* D2022), one of the dominant bacterial species in Douzhier, and evaluated its efficacy against influenza A virus (IAV) infection. **Methods:** A murine model of IAV infection was established using C57BL/6J mice. Following oral administration of *L. cremoris* D2022, treated and model control groups (IAV-infected model control group received phosphate-buffered saline (PBS)) were comparatively assessed through clinical outcomes (body weight dynamics and survival), pathological parameters (colon length, lung index, and lung histopathology), inflammatory cytokine profiling, multi-omics analyses (lung transcriptomics and cecal metabolomics), and adaptive immune responses. **Results:** Administration of *L. cremoris* D2022 markedly alleviated IAV-induced pathology compared with PBS-treated model controls, as evidenced by improved survival (30% vs. 0%, *p* = 0.006), reduced body weight loss, decreased lung index, and preservation of colon length. D2022 treatment accelerated viral clearance, accompanied by enhanced early interferon beta (IFN-β) production. This was followed by suppression of late-stage hyperinflammation, including reduced levels of interleukin-1β (IL-1β), interleukin-6 (IL-6), and tumor necrosis factor alpha (TNF-α) at 7 dpi. Metabolomic analysis revealed increased levels of deaminotyrosine (DAT), short-chain fatty acids (SCFAs), and microbially conjugated bile acids (e.g., ursodeoxycholic acid (UDCA) and isoallolithocholic acid (isoalloLCA)). Furthermore, IAV-specific adaptive immune responses were significantly enhanced. **Conclusions**: *L. cremoris* D2022 protects mice against IAV infection, with enhanced early IFN-β responses, reduced late-stage inflammation, and associated changes in intestinal microbial metabolites. The causal roles of these metabolites remain to be established. These findings support further evaluation of D2022 as a probiotic candidate for adjunctive influenza prevention.

## 1. Introduction

Douzhier is a traditional Chinese fermented food with a history spanning over 1000 years, dating back to the Song Dynasty. It possesses a distinctive flavor and remains especially popular among residents of Beijing. Douzhier is produced from mung beans through spontaneous fermentation [1,2]. Briefly, mung beans are first soaked in water, then ground and filtered to obtain a starch milk. This starch milk is inoculated with a portion of a previous batch of Douzhier and allowed to ferment. Upon standing, it separates into three layers according to density: the bottom layer consists mainly of mung bean starch; the middle layer, rich in mung bean protein, is known as madoufu (a traditional Beijing snack); and the top layer is a gray-green liquid, referred to as raw Douzhier [2,3]. The raw Douzhier is subsequently further fermented and cooked to yield the final product. Previous studies have shown that the fermentation process is accompanied by substantial changes in the diverse microbial community and fermentation-derived metabolites, including increased levels of free amino acids [1,3].

Seasonal influenza remains a major global public health burden, causing approximately 1 billion infections, 3–5 million cases of severe illness, and 290,000–650,000 respiratory deaths annually worldwide. In the United States, the 2024–2025 influenza season was associated with an estimated 51 million illnesses, 710,000 hospitalizations, and 45,000 deaths [4]. Influenza vaccines have been widely used for the prevention and control of influenza; however, their effectiveness is challenged by the virus’s high mutation rate, which enables varying degrees of immune evasion and reduced vaccine efficacy even when the vaccine strain is updated annually based on surveillance data [5]. Some small-molecule drugs have been successfully used to treat influenza infections for many years, but they are not suitable for long-term preventive use and are also at risk of developing drug resistance due to genetic mutations of the virus [6]. Therefore, novel methods for the prevention and treatment of influenza are warranted.

Previous studies suggest that the protective effects of certain probiotics against respiratory viral infections may involve host-directed mechanisms, including enhancement of type I interferon responses and attenuation of excessive inflammation [7,8,9]. Such mechanisms may be less directly affected by viral antigenic variation than antigen-specific immune responses. Accumulating evidence indicates that certain probiotics—such as *Lactococcus lactis* [10,11], *Lactobacillus plantarum* [12,13], *Leuconostoc mesenteroides* [14], *Akkermansia muciniphila* [7,15], and *Bacteroides fragilis* [16]—exhibit anti-influenza activity. Current studies suggest that these effects are largely mediated through antigen-independent mechanisms, including the enhancement of type I interferon responses and the attenuation of excessive inflammation. So, such mechanisms are less susceptible to viral genetic variation. Moreover, many probiotics can be incorporated into fermented foods, which enables long-term dietary intake of probiotics and offers a practical strategy for sustained protection against influenza.

As mentioned above, Chinese Douzhier harbors a complex microbial ecosystem in which *Lactococcus lactis* subsp. *cremoris* (*L. cremoris*) represents the dominant bacterial species [2,3]. This organism is extensively applied in food fermentation and serves as a principal probiotic constituent of some well-known traditional fermented products such as kefir and Caspian Sea yogurt, both originating from the Caucasus region [17]. Importantly, probiotic activity is highly strain-dependent, underscoring the functional heterogeneity within *L. cremoris*. Previous studies have demonstrated that distinct strains can differentially regulate host immunity, preserve gut microbial homeostasis, and suppress respiratory virus replication, including influenza and parainfluenza viruses [8,18]. Supporting this concept, *L. cremoris* YRC3780 isolated from *Kefir* alleviates allergic inflammation and reduces Japanese cedar pollinosis in murine models [19]. In parallel, *L. cremoris* FC derived from *Caspian Sea yogurt* confers protection against influenza virus infection, as evidenced by improved survival, reduced lung viral burden, and attenuated pulmonary pathology [11].

In the present study, we isolated a novel strain of *L. cremoris* from Chinese Douzhier, designated *L. cremoris* D2022. However, whether *L. cremoris* originating from Chinese Douzhier exhibits comparable or distinct antiviral properties remains unclear. In particular, their potential role in modulating host responses to influenza virus infection has not been investigated. The primary aim of this study was to determine whether *L. cremoris* D2022 isolated from Douzhier confers protection against influenza A virus (IAV) infection in mice and to investigate the biological mechanisms associated with this protection, with particular emphasis on antiviral interferon responses, inflammatory regulation, gut microbial metabolites, and adaptive immunity.

## 2. Materials and Methods

### 2.1. Animals and Ethics Statement

All mice were purchased from Beijing Vital River Laboratory Animal Technology Co., Ltd., Beijing, China (Certificate No.: SCXK (Beijing) 2012-0001) and housed in the Experiment Animal Center of the Chinese Center for Disease Control and Prevention. The animal procedures used in this study were all approved by the Welfare and Ethical Inspection at the Animal Experiment Center of Chinese Center for Disease Control and Prevention (Approval number 2024-033).

### 2.2. Culture of Influenza Virus and L. cremoris D2022

Influenza virus A/Puerto Rico/8/34 mouse-lung-adaptive strain (PR8) was kindly provided by the Chinese National Influenza Center, National Institute for Viral Disease Control and Prevention, Chinese Center for Disease Control and Prevention. PR8 was reproduced with Madin-Darby canine kidney (MDCK) cells and quantified using a plaque assay as previously described [20].

Douzhier samples were serially diluted ten-fold, and 100 μL of each dilution was spread onto Brain Heart Infusion (BHI) sheep blood agar plates. Following incubation at 37 °C for 24 h, colonies were counted, and viable bacterial numbers were expressed as colony-forming units (CFU)/mL.

*L. cremoris* D2022, was isolated from *Douzhier*. The isolation process has been detailed previously [21]. The quercetin-degrading activity of the strain was assessed as previously described [22], and whole-genome sequencing data were analyzed. D2022 was identified as a novel strain of *Lactococcus cremoris*. The genome sequencing data of this novel strain was submitted to NCBI Genbank (BioProject No. PRJNA1014919).

### 2.3. Mouse Experiment Design

A total of 120 6-week-old female C57BL/6J mice were used in four independent animal experiments. Each experiment included 30 mice, which were randomly allocated to three groups (*n* = 10 per group): a non-infected control group (Control), an influenza A virus (IAV)-infected group treated with phosphate-buffered saline (PR8 + PBS), and an IAV-infected group treated with Lactococcus cremoris D2022 (PR8 + D2022). PBS was used as the vehicle control to match the gavage procedure, administration volume, and treatment frequency of the D2022-treated group.

For the infection groups, mice were anesthetized with an intraperitoneal injection of 1% pentobarbital and then intranasally inoculated with either a non-lethal (450 plaque-forming units [PFU]) or lethal dose (900 PFU) of influenza virus H1N1 PR8 [22], with the infection day designated as day 0. Mice in the infection groups received daily oral administration of 200 µL of *L. cremoris* D2022 (2 × 10^9^ CFU) or PBS from day −5 to day 7.

In Experiments 1–3, mice in the infection groups were challenged intranasally with a non-lethal dose of PR8 (450 PFU). Experiment 1 was terminated at 1 dpi, Experiment 2 at 3 dpi, and Experiment 3 at 7 dpi for the corresponding sample collection and analyses. In Experiment 4, mice in the infection groups were challenged with a lethal dose of PR8 (900 PFU), and survival was monitored daily through 14 dpi. Each experiment included 10 mice in the control group, 10 mice in the PR8 + PBS group, and 10 mice in the PR8 + D2022 group.

Throughout the study, mice were housed in groups of five per cage with free access to food and water under a 12-h light/dark cycle. At the end of the experiments, surviving mice were fasted for 6 h, anesthetized with an intraperitoneal injection of 1% pentobarbital sodium (50 mg/kg), and then sacrificed by cervical dislocation.

### 2.4. Measurements for Lung Index, Colon Length, Body Weight Loss and Viral Load of Influenza Virus

All mice were monitored daily for body weight and sacrificed on days 1, 3, and 7 post-infection. Colon and whole-lung specimens were collected to determine colon length and lung index [calculated as lung weight (g)/body weight (g) × 100%] [23]. After measuring the lung index, Lung tissues were homogenized using a tissue grinder (TissueLyser II, QIAGEN, Hilden, Germany) and centrifuged at 12,000 rpm for 10 min. The resulting supernatant was collected for influenza virus RNA extraction using the QIAamp Viral RNA Mini Kit (QIAGEN GmbH, Lot. 163014099). Viral loads in the lungs were quantified using a commercial influenza A virus nucleic acid testing kit (Jiangsu Hechuang Biotechnology Co., Ltd., Zhenjiang, China) on an ABI 7500 Fast Real-Time PCR System (ABI, Foster City, CA, USA), following the manufacturer’s instructions.

### 2.5. Lung Histopathological Examination

Lung tissues were fixed in 10% formalin for 24 h, embedded in paraffin, and sectioned at a thickness of 5 μm. The sections were stained with hematoxylin and eosin (H&E) and evaluated independently by two experienced pathologists using light microscopy. Histopathological changes were scored on a scale of 1 to 4 based on the lung histopathological scoring criteria described by Mann et al. [24].

### 2.6. Measurement of Cytokine Profiles in the Serum and Lung Tissues on Day 3 and Day 7

A total of eight cytokines and chemokines [interleukin (IL)-1β, IL-6, tumor necrosis factor (TNF)- α, keratinocyte chemoattractant (KC), IL-4, IL-10, IL-12, and IFN-γ] in the serum and lung tissue homogenates were measured using a LabEx Biomarker Mouse 8-Plex kit (RD) on a Luminex 200 (Luminex 200, Luminex Corporation, Austin, TX, USA) system in accordance with the manufacturers’ instructions.

### 2.7. Transcriptome Analysis of the Lungs

Total RNA was extracted from the lung tissues of mice using TRIzol reagent, following the manufacturer’s instructions. RNA concentration was quantified, and its integrity was assessed by fragment analysis using an Agilent 2100 Bioanalyzer (Thermo Fisher Scientific, Waltham, MA, USA). Qualified RNA samples were used for library construction and RNA sequencing on the BGISEQ-500 platform by Shenzhen BGI Genomics Co., Ltd. (Shenzhen, China). Raw data were processed using SOAPnuke (version 1.5.2) to remove adapter-containing sequences, generating clean data. Alignment of more than 10% of N-containing reads to the reference genes and genome was performed using Bowtie2 and HISAT, respectively. The matched reads were normalized to fragments per kilobase million (FPKM) using RSEM for gene expression analysis. Gene set enrichment analysis (GSEA) was performed with parameters nperm = 1000, min = 15, and max = 500 using GSEA software (version 3.0). The original data were deposited in the NCBI SRA database (accession number: PRJNA1100256 for day 1; PRJNA1102099 for day 7).

### 2.8. Coculture of mBMDCs of Mice and L. cremoris D2022

Mouse bone marrow cells were isolated from the femurs and tibias of 4–6-week-old C57BL/6J mice and cultured according to the protocol established by Inaba et al. [25]. Briefly, cells were cultured in RPMI 1640 medium (Luminex 200, Luminex Corporation, Austin, TX, USA) supplemented with 10% fetal calf serum (Gibco), 10 ng/mL of mouse granulocyte-macrophage colony-stimulating factor (GM-CSF) (Hangzhou Kaihua Biotechnology Co., Ltd., Hangzhou, China), and 10 ng/mL of mouse IL-4 (PeproTech, Cranbury, NJ, USA). The culture medium was fully replaced with fresh medium on day 3, and half of the medium was replaced on day 5. On day 7, suspended immature mouse bone marrow-derived dendritic cells (mBMDCs) were collected and purified using MojoSort™ Mouse CD11c Nanobeads (BioLegend, San Diego, CA, USA) following the manufacturer’s instructions. The purified mBMDCs were then co-cultured with *L. cremoris* D2022 at bacteria-to-cell ratio of 10:1 for 24 h to evaluate the in vitro effects of *L. cremoris* D2022, with lipopolysaccharide (LPS) serving as a positive control.

### 2.9. The Expression of IFN-β Assay

The expression of IFN-β in lung tissue was assessed on days 1, 3, and 7. Additionally, the concentrations of IFN-β in the supernatants of cultured cells were measured. These detections were performed using an ELISA kit (Invitrogen, Carlsbad, CA, USA), following the manufacturer’s instructions.

### 2.10. Western Blotting

Tissue homogenates were lysed using RIPA Buffer (Solarbio, CN, Beijing, China) on ice. Proteins were extracted from the supernatants of tissue lysates using methanol and chloroform, following the method described by Luo et al. [26]. The proteins were then separated by SDS-PAGE and transferred onto a nitrocellulose membrane (Cytiva, Little Chalfont, UK). After blocking with PBS containing 10% FBS, the membranes were incubated overnight at 4 °C with primary antibodies against P65 (1:1000, Cell Signaling Technology, Danvers, MA, USA), p-P65 (1:1000, Cell Signaling Technology, Danvers, MA, USA), IκBα (1:1000, Cell Signaling Technology, USA), p-IκBα (1:1000, Cell Signaling Technology, Danvers, MA, USA), and β-actin (1:1000, LabLead, CN, Beijing, China). Following washing with TBST, the membranes were incubated with rabbit anti-mouse IgG secondary antibodies at room temperature for 1 h. Protein bands were visualized using a Western blot system (Solarbio, CN), and the band intensities were quantified using ImageJ 1.52v and normalized to β-actin.

### 2.11. Metabolomic Analysis

Metabolomic analyses of the cecal contents from mice were performed at BGI Genomics Co., Ltd. (Wuhan, China) using a UPLC I-Class Plus system (Waters, Milford, MA, USA) coupled with a QTRAP 6500 Plus mass spectrometer (SCIEX, Marlborough, MA, USA) for the quantification of over 700 small metabolite molecules (HM700-targeted quantification). Raw LC-MS/MS data were processed to extract and identify peaks, generating metabolite peak areas and identification results. Data preprocessing was performed using MetaX to derive compound intensities and forms for subsequent analysis. Identified metabolites were classified and annotated by comparing them with the KEGG and HMDB databases.

### 2.12. Splenocyte Being Analyzed with Flow Cytometry

Mouse splenocytes (1 × 10^6^ cells/well) were isolated and stimulated with Cell Activation Cocktail (2 µL/mL, Biolegend, San Diego, CA, USA) at 37 °C and 5% CO_2_ for 4 h. The activated cells were harvested by centrifugation at 350 ×g for 5 min. After discarding the supernatant, anti-CD16/CD32 antibody (Biolegend) was added to block non-specific immunofluorescent staining. For cell surface staining, splenocytes were incubated with anti-CD3, anti-CD4, and anti-CD8 surface marker antibodies (Biolegend) for 20 min at 4 °C. For intracellular staining, cells were washed with Staining Buffer (Biolegend) and resuspended in Fix/Perm Buffer (Biolegend) for 30 min at room temperature to enable permeabilization. Subsequently, cells were incubated with anti-IFN-γ and anti-IL-4 antibodies (Biolegend) for 20 min and washed with Perm Wash solution. Finally, all samples were analyzed using a FACSCalibur flow cytometer (BD, Franklin Lakes, NJ, USA).

### 2.13. Influenza-Specific IFN-γ Secreting Splenocyte Cells Assay

The adaptive T-cell response to influenza strain PR8 HA peptides was evaluated using a mouse IFN-γ ELISpot assay kit (BD Biosciences), following the manufacturer’s guidelines. First, 96-well plates were coated overnight at 4 °C with anti-mouse IFN-γ antibodies (1:250, 100 µL per well). After three washes with PBS, the plates were blocked with 200 µL of RPMI 1640 medium containing 10% fetal bovine serum for 2 h at room temperature. A suspension of mouse splenocytes (1 × 10^6^ cells/well) was added to the coated wells and stimulated with HA peptides (5 µg/mL, ChinaPeptides Co., Ltd., Shanghai, China) at 37 °C in 5% CO_2_ for 20 h. Following this, the plates were washed three times with PBS containing Tween 20 (PBST). Biotinylated anti-mouse IFN-γ antibody (1:200) was then added and incubated for 2 h at room temperature, followed by five washes with PBST. Streptavidin-conjugated horseradish peroxidase (HRP; 1:100) was added and incubated for 1 h at room temperature. After adding 100 µL of substrate to stain the spots, the plates were washed with distilled water and air-dried in the dark. Finally, the spot-forming cells were counted using the iSpot Spectrum (AID, Strassberg, Germany) according to the manufacturer’s instructions.

### 2.14. Statistical Analysis

Continuous variables are presented as means ± standard deviation, and categorical variables are expressed as counts (percentages). Comparisons of continuous variables between groups were made using one-way analysis of variance (ANOVA) or the Kruskal–Wallis rank-sum test, as appropriate. Dunnett’s test was applied for multiple comparisons. Survival among the groups was analyzed using the Kaplan–Meier method and compared with the log-rank test. All statistical analyses were performed using GraphPad Prism 9.0 (GraphPad Software, San Diego, CA, USA). A *p* value < 0.05 was considered statistically significant.

## 3. Results

### 3.1. Microbiological Characteristics of L. cremoris D2022

*L. cremoris* D2022 was isolated from Douzhier, which contained approximately 3 × 10^7^ CFU/mL of bacteria with *L. cremoris* D2022 as the predominant strain. *L. cremoris* D2022 is a Gram-positive bacterium that exhibited strong quercetin-degrading activity, with a degradation rate of approximately 65% (Figure S2).

Whole-genome sequencing analysis of *L. cremoris* D2022 revealed that the D2022 genome is approximately 2.63 Mb in size with a GC content of 35.42%. Additional functional analyses were performed with particular attention to genes associated with bile acid metabolism, which are relevant to the metabolic phenotypes investigated in this study.

In addition, we compared D2022 with representative publicly available *L. cremoris* genomes. Whole-genome phylogenetic analysis and average nucleotide identity (ANI) analysis were performed to determine the genomic relationships between D2022 and other *L. cremoris* strains. Genome characteristics and ANI values are summarized in Table S1, while the phylogenomic tree and ANI heatmap are presented in Figure S1. Selected genes related to bile acid metabolism were further compared among these strains and are summarized in Table S2.

With respect to bile acid metabolism, a candidate bile salt hydrolase (BSH)-family homolog was detected in D2022. This protein showed extensive query coverage but relatively low amino acid sequence identity to the experimentally characterized BSH reference and was therefore conservatively annotated as a candidate homolog rather than as a functionally confirmed BSH. In contrast, no significant homolog of the canonical bile acid 7α-dehydratase BaiE was detected in D2022 under the predefined homology-search criteria.

### 3.2. L. cremoris D2022 Improves Survival, Relieves Inflammation in Lung and Colon, and Reduces Viral Load in Lung of IAV-Infected Mice

**Figure 1:**
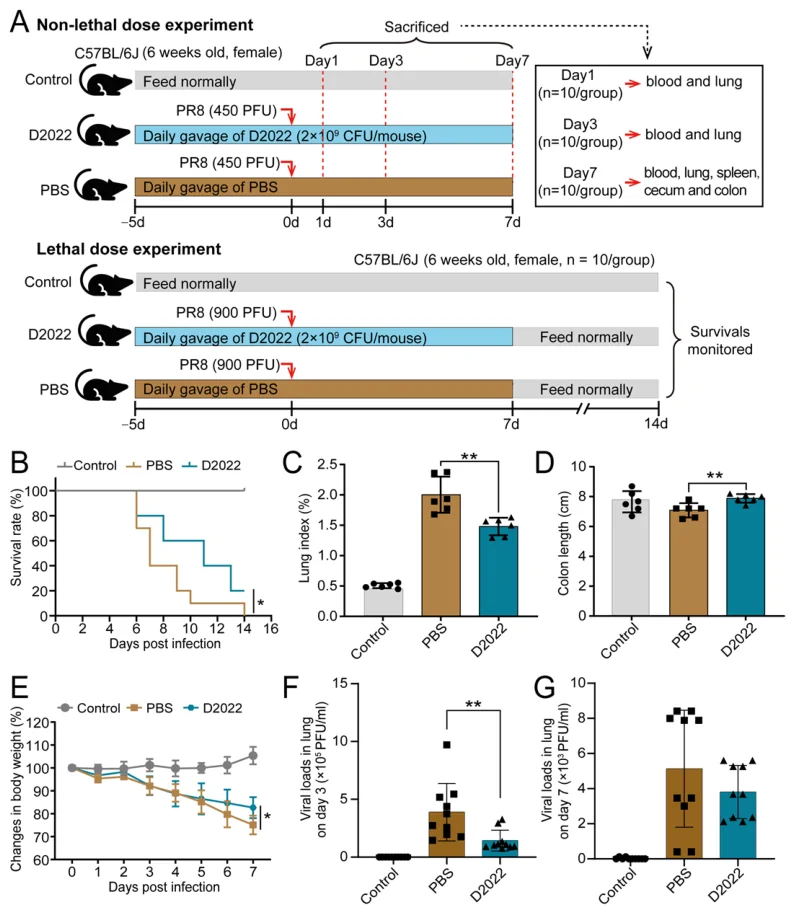
Experimental design and protective effects of D2022 against influenza. (**A**) Schematic overview of the four independent animal experiments. A total of 120 female C57BL/6J mice were used. Each experiment included 30 mice randomly assigned to the control, PR8 + PBS, and PR8 + D2022 groups (*n* = 10 per group). Experiments 1–3 used a non-lethal PR8 challenge (450 PFU) and were terminated at 1, 3, and 7 dpi, respectively. Experiment 4 used a lethal PR8 challenge (900 PFU), and survival was monitored through 14 dpi. (**B**) Survival of the three groups of mice within 14 days post-infection. (**C**) Lung index on day 7. (**D**) Colon length on day 7. (**E**) Temporal trends of body weights of mice in each group 7 days after infection. (**F**) Viral load in the lung on day 3. (**G**) Viral load in the lung on day 7. Data in panels (**C**,**D**,**F**,**G**) are presented as mean ± standard deviation from three independent experiments. Data shown in panels (**B**,**E**) were obtained from one of three independent experiments. * *p* < 0.05, ** *p* < 0.01.

Upon exposure to a non-lethal dose of influenza virus, mice treated with *L. cremoris* D2022 exhibited significant improvements in multiple physiological parameters on day 7 post-infection, including lung index (1.48 ± 0.16 vs. 2.06 ± 0.32), colon length (7.78 ± 0.17 cm vs. 6.82 ± 0.20 cm), and body weight (83.6 ± 5.22% vs. 75.3 ± 4.66%) compared to PBS-treated controls (Figure 1C–E).

D2022-treated mice also showed significantly lower viral loads in the lungs at 3 days post-infection (396,000 ± 220,000 PFU vs. 146,000 ± 56,000 PFU) relative to PBS-treated mice. However, by day 7 post-infection, no significant differences were observed between the two groups (4800 ± 3600 PFU vs. 3700 ± 1800 PFU, respectively) (Figure 1F–G).

Following challenge with a lethal dose of influenza virus, all PBS-treated mice (*n* = 10) succumbed to infection within 13 days post-infection. In contrast, 3 out of 10 D2022-treated mice survived until the endpoint at 14 days post-infection (Figure 1B).

### 3.3. L. cremoris D2022 Alleviated Lung Pathological Injury of IAV-Infected Mice

**Figure 2:**
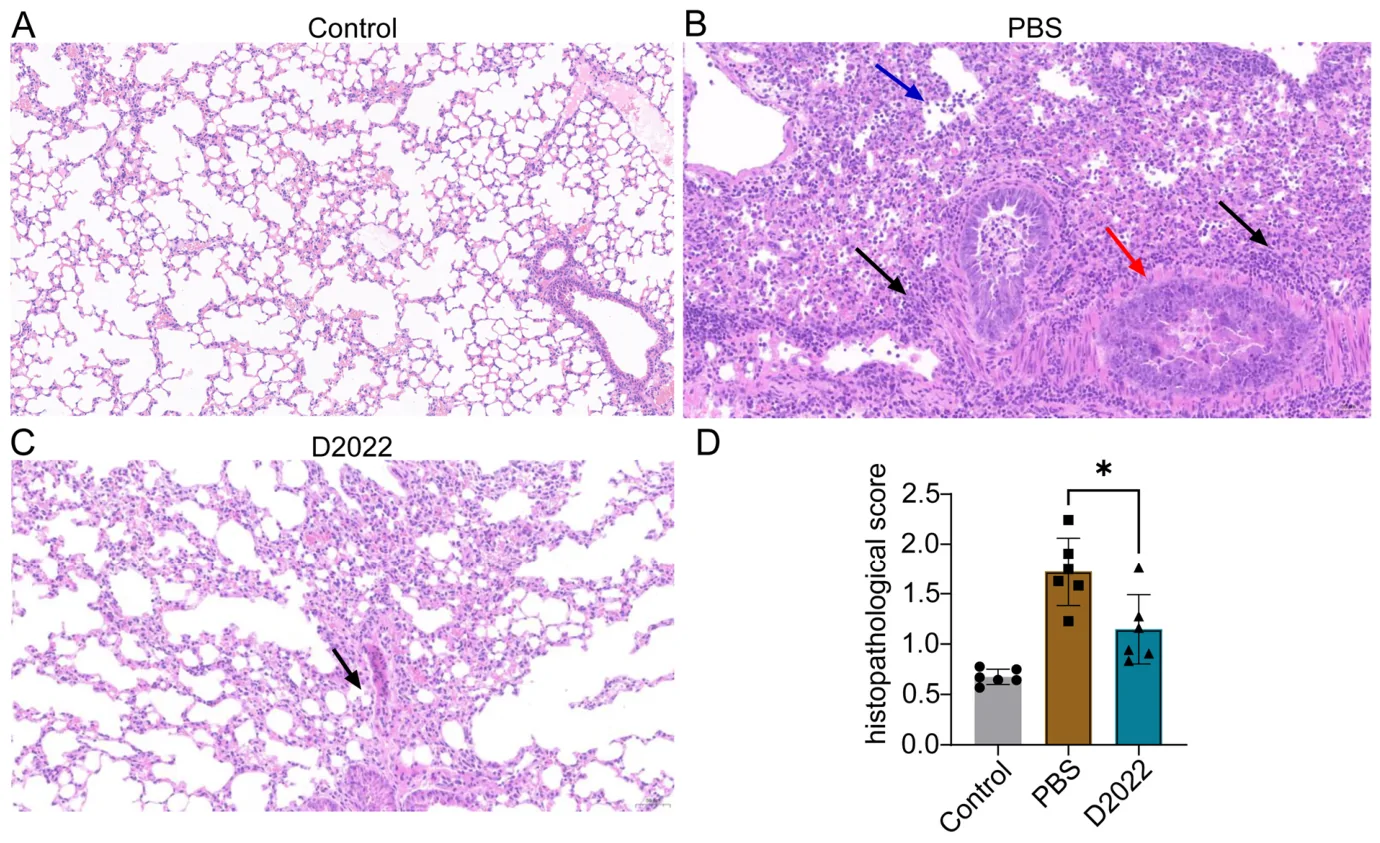
Histopathological findings in the lungs of mice 7 days post-infection. (**A**–**C**) Lung histopathological findings (hematoxylin and eosin staining, 200× magnification) of the control group, PBS group, and D2022 group. Inflammatory cell infiltration in the alveoli (black arrow), edema around the blood vessels, loose connective tissue arrangement in the alveoli (blue arrow), bronchial epithelial cell necrosis, and nuclear fragmentation (red arrow) after infection are evident. (**D**) Histopathological scores of the three groups. Representative results are shown from one of three independent experiments. * *p* < 0.05.

*L. cremoris* D2022 treatment markedly alleviated pathological injuries in the lungs of IAV-infected mice (Figure 2A–D). The alveolar wall thickening was reversed (green arrows, Figure 2), accompanied by a decrease in inflammatory cell infiltration (black arrows, Figure 2). Improved edema around the blood vessels was observed, along with a looser connective tissue arrangement in the alveoli (blue arrows, Figure 2), attenuation of bronchial epithelial cell necrosis and nuclear fragmentation (red arrows, Figure 2). The lung pathological scores of the infected mice treated with *L. cremoris* D2022 (1.26 ± 0.42) were significantly lower than those of the PBS group (1.72 ± 0.38) (Figure 2D).

### 3.4. Effects of L. cremoris D2022 on Cytokines in the Lungs and Serum of IAV-Infected Mice

**Figure 3:**
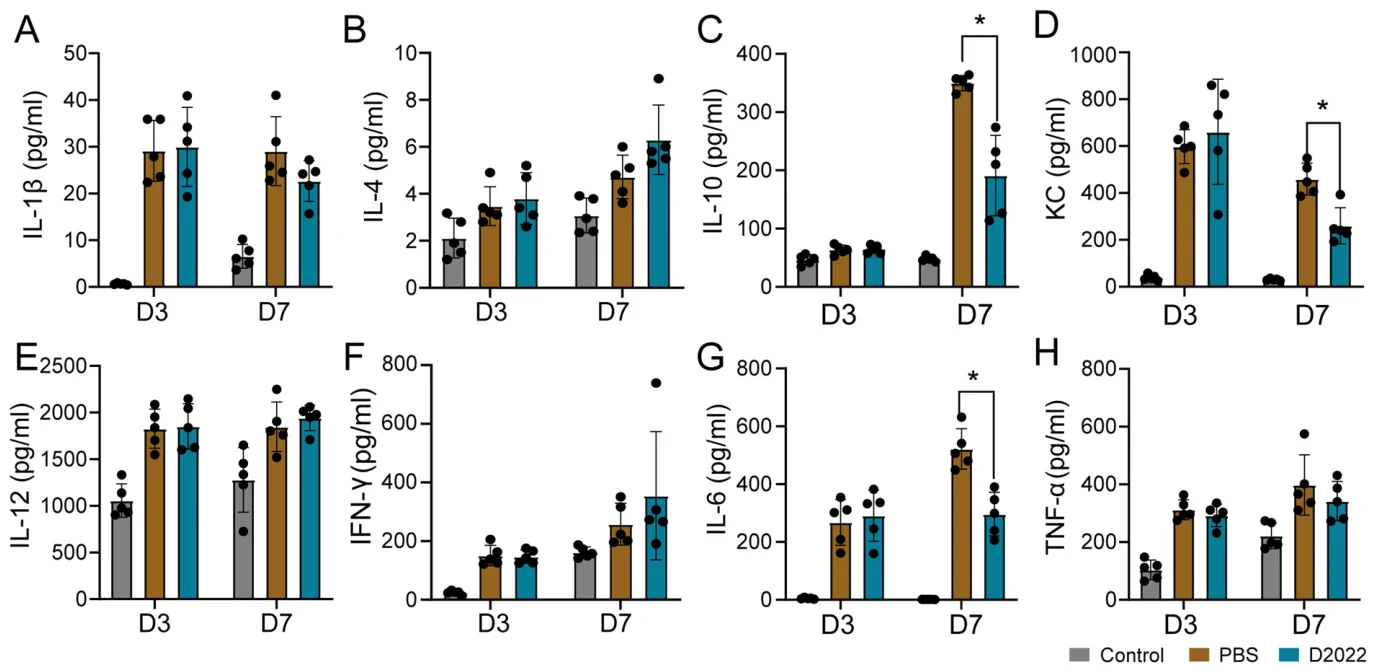
Effect of administration of D2022 on the lung cytokine profile on day 3 and day 7 post-infection. Levels of cytokines and chemokines in the lung of the three groups of mice: (**A**) IL-1β, (**B**) IL-4, (**C**) IL-10, (**D**) KC, (**E**) IL-12, (**F**) IFN-γ, (**G**) IL-6, and (**H**) TNF-α. Data are presented as mean ± standard deviation of three independent experiments. * *p* < 0.05.

The levels of multiple cytokines and chemokines in the lungs of influenza virus (IAV)-infected mice were significantly elevated on days 3 and 7 post-infection compared to the non-infected group. These included IL-1β (Figure 3A), IL-4 (Figure 3B), IL-10 (Figure 3C), KC (CXCL1; Figure 3D), IL-12 (Figure 3E), IFN-γ (Figure 3F), IL-6 (Figure 3G), and TNF-α (Figure 3H).

*L. cremoris* D2022 treatment had minimal impact on cytokine and chemokine levels on day 3. However, by day 7, it resulted in a reduction in proinflammatory mediators in the lungs, with statistically significant decreases observed in IL-6 and KC, and non-significant reductions in IL-1β and TNF-α. Interestingly, *L. cremoris* D2022 also led to a notable decrease in the anti-inflammatory cytokine IL-10. Furthermore, levels of adaptive immune-associated cytokines, including IL-4, IL-12, and IFN-γ, were increased in the D2022-treated group on day 7, although these changes all were not with statistical significance.

**Figure 4:**
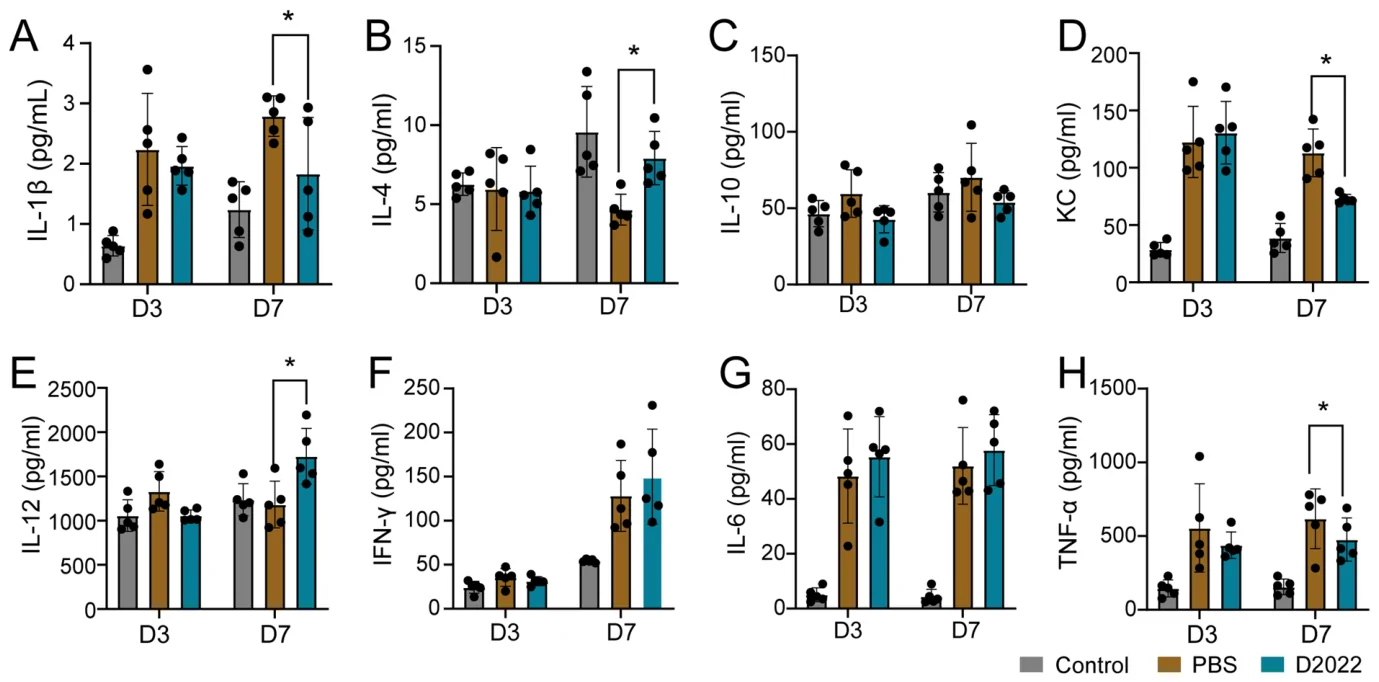
Effects of administration of D2022 on the serum cytokine profiles on day 3 and day 7 post-infection. Serum levels of cytokines and chemokines in the three groups: (**A**) IL-1β, (**B**) IL-4, (**C**) IL-10, (**D**) KC, (**E**) IL-12, (**F**) IFN-γ, (**G**) IL-6, and (**H**) TNF-α. Data are presented as mean ± standard deviation of three independent experiments. * *p* < 0.05.

A similar trend in cytokine modulation was observed in serum samples (Figure 4A–H). On day 7, *L. cremoris* D2022 treatment significantly decreased the serum levels of proinflammatory cytokines and chemokines (IL-1β, KC, and TNF-α), while promoting an increase in adaptive immunity-related cytokines (IL-12 and IFN-γ).

### 3.5. Lung Transcriptome Analyses Suggested That L. cremoris D2022 Up- or Down-Regulated Multiple Signal Pathways of IAV-Infected Mice

**Figure 5:**
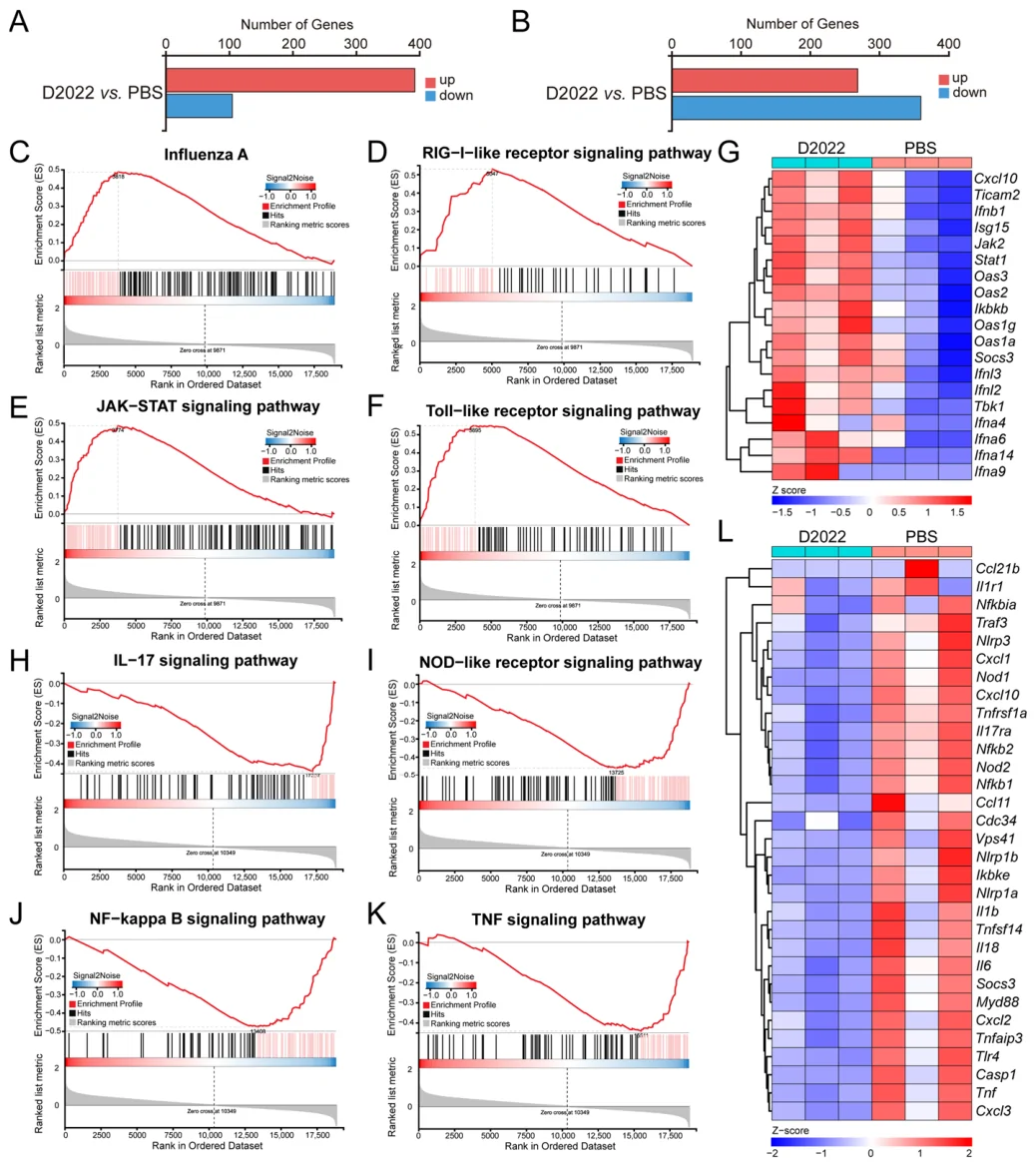
Transcriptome analyses of the lung tissues of the mice. (**A**) Up- and down-regulated genes in the D2022 group compared with the PBS group on day 1. (**B**) Up- and down-regulated genes in the D2022 group compared with the PBS group on day 7. (**C**–**F**) Up-regulated signal pathways in the D2022 group identified by gene set enrichment analysis (GSEA) compared with the PBS group on day 1. (**G**) Heatmap showing differential expression of genes related to the interferon regulatory factors (up-regulated genes) between PBS group and D2022 group on day 1. (**H**–**K**) Down-regulated signal pathways in the D2022 group identified by gene set enrichment analysis (GSEA) compared with the PBS group on day 7. (**L**) Heatmap showing differential expression of genes related to cytokines and chemokines (down-regulated genes) between PBS group and D2022 group on day 7.

A total of 495 differentially expressed genes (DEGs) were identified in the D2022-treated group compared to the PBS-treated group on day 1, with 391 genes up-regulated and 104 genes down-regulated in the D2022 group. On day 7, 627 DEGs were identified, with 268 genes up-regulated and 359 genes down-regulated in the D2022 group (Figure 5A,B).

KEGG enrichment analysis revealed that, on day 1, the majority of the DEGs were up-regulated, whereas, on day 7, most of them were down-regulated. Gene Set Enrichment Analysis (GSEA) of the DEGs on day 1 identified four up-regulated pathways, including the Influenza proliferation process, *RIG-I*-like receptor signaling pathway, Toll-like receptor signaling pathway, and *JAK-STAT* signaling pathway (Figure 5C–F). These pathways are primarily associated with *IFN*-related antiviral responses, and several key signaling molecules were up-regulated, such as interferon regulatory factors (*IRF1* and *IRF7*), interferons (*IFN-α*, *IFN-β*, and *IFN-γ*), and antiviral proteins (*ISGs* and *OAS*) (Figure 5G). On day 7, GSEA revealed four down-regulated pathways, including the *IL-17* signaling pathway, *NF-κB* signaling pathway, *NOD*-like receptor signaling pathway, and TNF signaling pathway (Figure 5H–K), which are related to anti-inflammatory mechanisms. Correspondingly, genes involved in inflammation and apoptosis were down-regulated, such as pro-apoptotic cytokines (Fas and Casp4), pro-inflammatory cytokines (e.g., *IL-1β*, *IL-6*, *TNF-α*, *IL-17a*, *CCL4*, and *CXCL10*), Toll-like receptors (*TLR4*), NOD-like receptors (e.g., *NLRP1b*, *NLRP3*, *NLRC4*, and *NLRP12*), acute lung injury-related cytokines (*S100a9*), and co-stimulatory factors (*CD40*) (Figure 5L).

**Figure 6:**
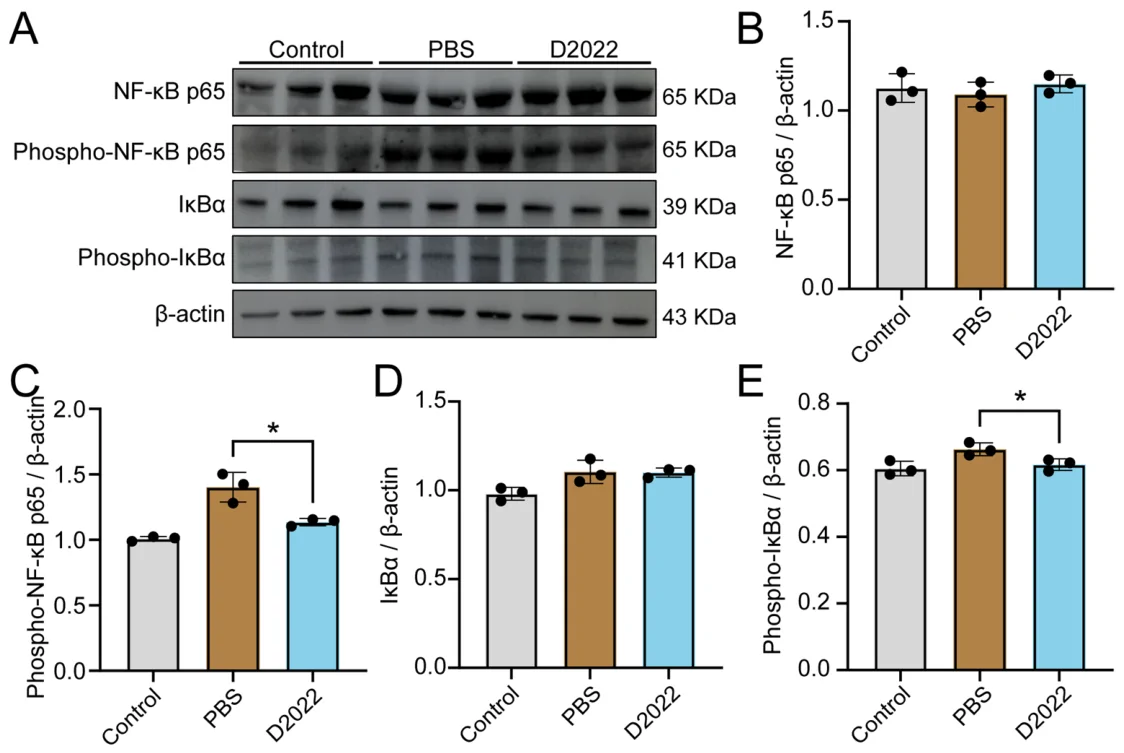
Effect of D2022 on NF-κB pathway related protein expression in lung. (**A**) Western blot analysis of NF-κB pathway related protein expression, (**B**) NF-κB p65 protein levels, (**C**) phospho-NF-κB p65 protein levels, (**D**) IκBα protein expression, and (**E**) phospho-IκBα protein levels. Representative results are shown from one of three independent experiments. * *p* < 0.05.

Additionally, we compared the expression of key signaling molecules in the *NF-κB* pathway between the PBS and D2022 groups at 7 days post-infection. We found that *L. cremoris* D2022 treatment reduced the levels of Phospho-NF-κB p65 and Phospho-IκBα proteins (Figure 6A–E), which was consistent with the transcriptomic findings.

### 3.6. L. cremoris D2022 Increased the Expression of IFN-β of mBMDCs and IAV-Infected Mice

**Figure 7:**
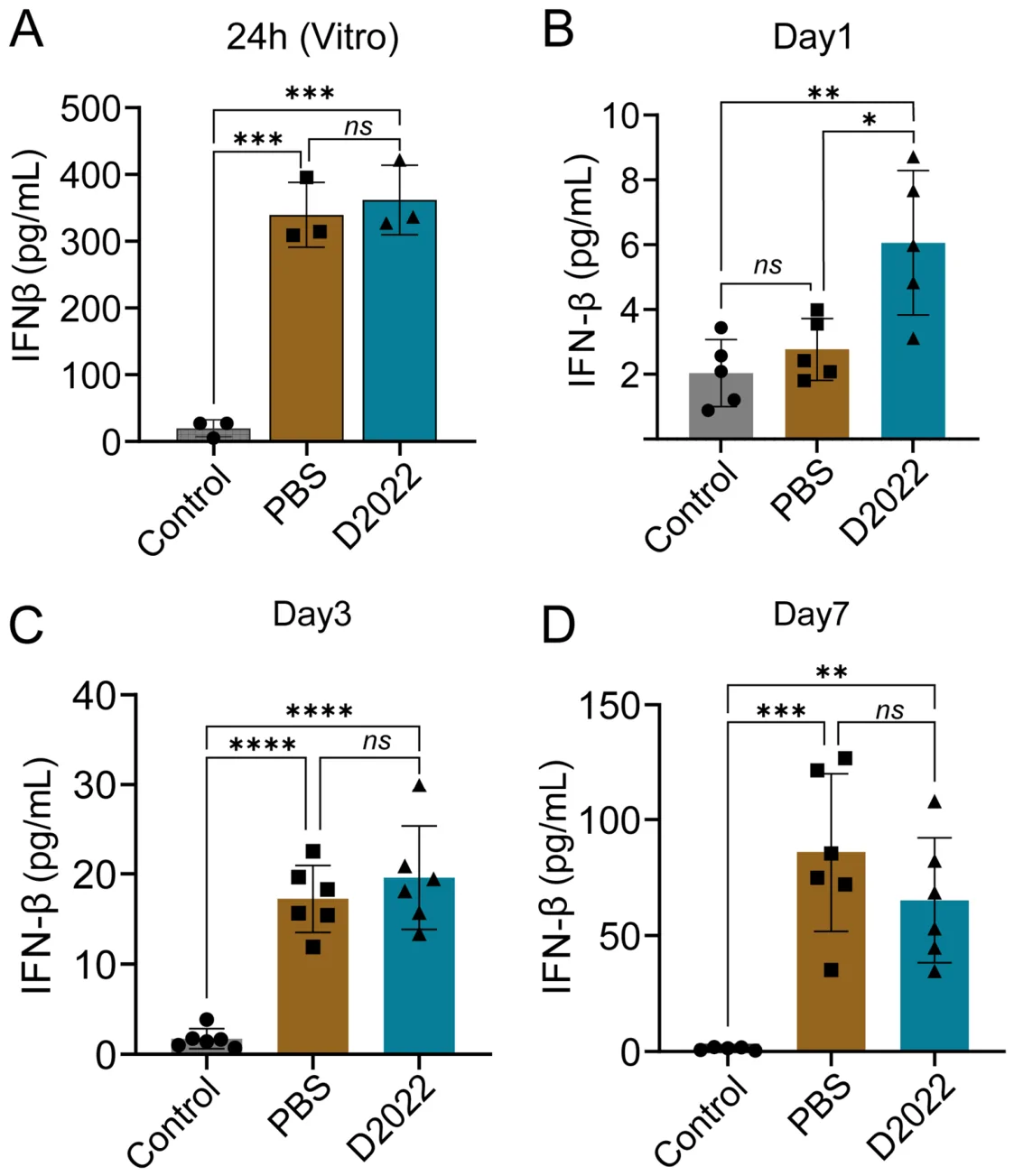
D2022 enhances the antiviral function via IFN-β. The IFN-β protein expressions of mBMDCs were detected by ELISA after incubation with D2022; LPS is a positive group (**A**). Gene relative expression levels of IFN-β in lung tissue of mice were measured on day 1 (**B**), day 3 (**C**) and day 7 postinfection (**D**) by ELISA after incubation with D2022. * *p* < 0.05; ** *p* < 0.01; *** *p* < 0.001; **** *p* < 0.0001; ns, not significant.

*L. cremoris* D2022 treatment increased the expression of IFN-β in mBMDCs when co-cultured with *L. cremoris* D2022 (Figure 7A). Dynamic comparison showed that the level of IFN-β in the lung tissue of the D2022 group rose more rapidly and was significantly higher than that in the PBS group at 1 dpi. However, the difference in IFN-β levels between the two groups was no longer statistically significant at 3 and 7 dpi (Figure 7B–D).

### 3.7. Metabolome Analyses Suggested That L. cremoris D2022 Increased Multiple Beneficial Metabolite Levels (SFCA, Secondary BA and DAT) in Intestine of IAV-Infected Mice

**Figure 8:**
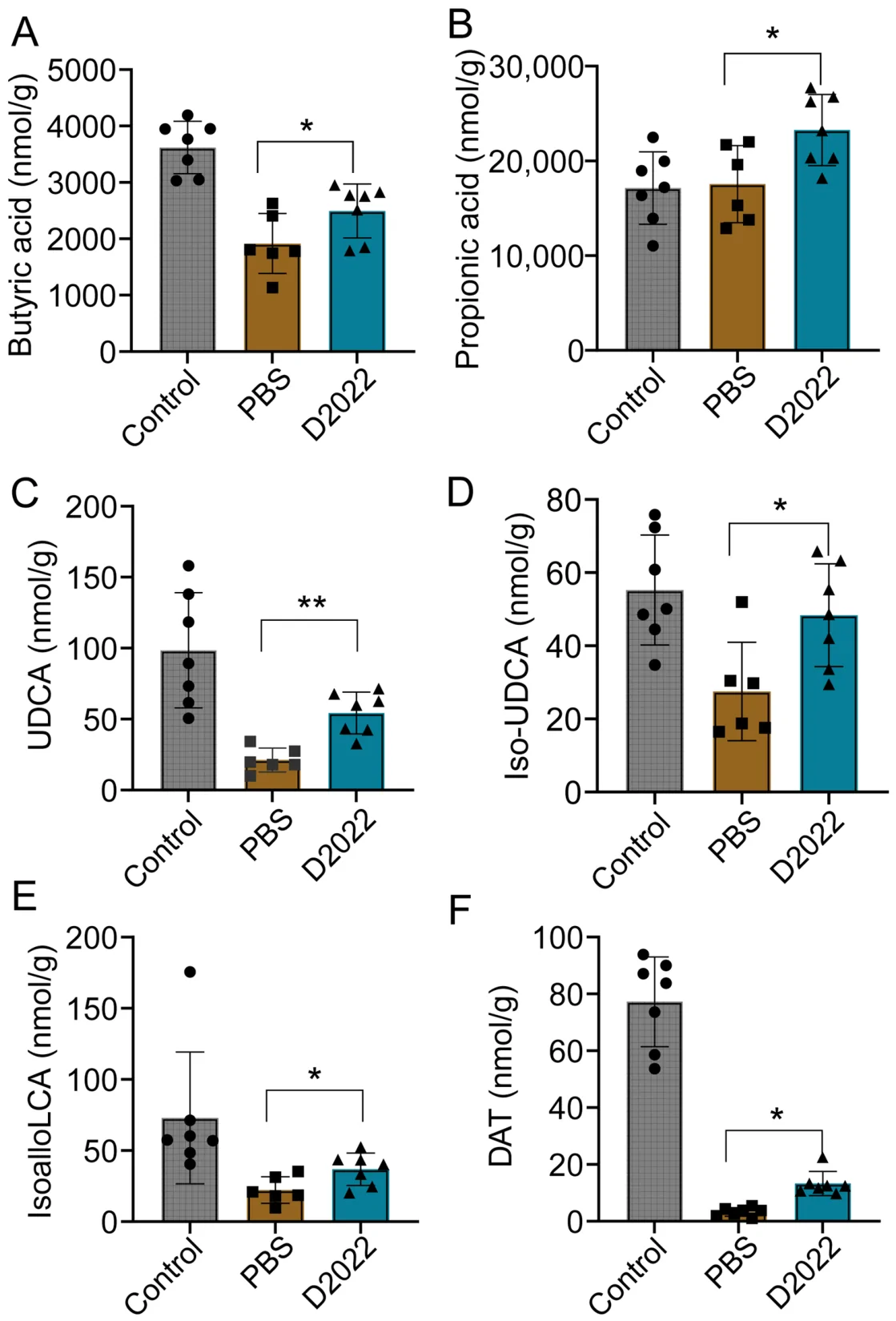
D2022 may inhibit inflammatory response by regulating SCFA and BA metabolism. (**A**) Concentration of butyric acid in the gut. (**B**) Concentration of Propionic acid in the gut. (**C**) Concentration of UDCA in the gut. (**D**) Concentration of iso-UDCA in the gut. (**E**) Concentration of isoalloLCA in the gut. (**F**) Concentration of DAT in the gut. * *p* < 0.05; ** *p* < 0.01.

To further investigate the effects of *L. cremoris* D2022 on intestinal metabolites in IAV-infected mice, we conducted a metabolomics analysis of cecal contents, focusing on metabolites derived from the gut microbiota. Compared to the non-infected group, influenza infection significantly reduced the levels of short-chain fatty acids (SCFAs), such as butyric acid (Figure 8A) and propionic acid (Figure 8B), as well as the production of secondary bile acids (BAs), including ursodeoxycholic acid (UDCA) (Figure 8C), Iso-UDCA (Figure 8D), and Isoallolithocholic acid (IsoalloLCA) (Figure 8E). Influenza infection also markedly lowered the level of Desaminotyrosine (DAT) (Figure 8F). However, *L. cremoris* D2022 treatment successfully reversed these reductions in SCFAs, secondary BAs (particularly UDCA), and DAT.

### 3.8. L. cremoris D2022 Accelerated the Adaptive Cellular Immune Response

**Figure 9:**
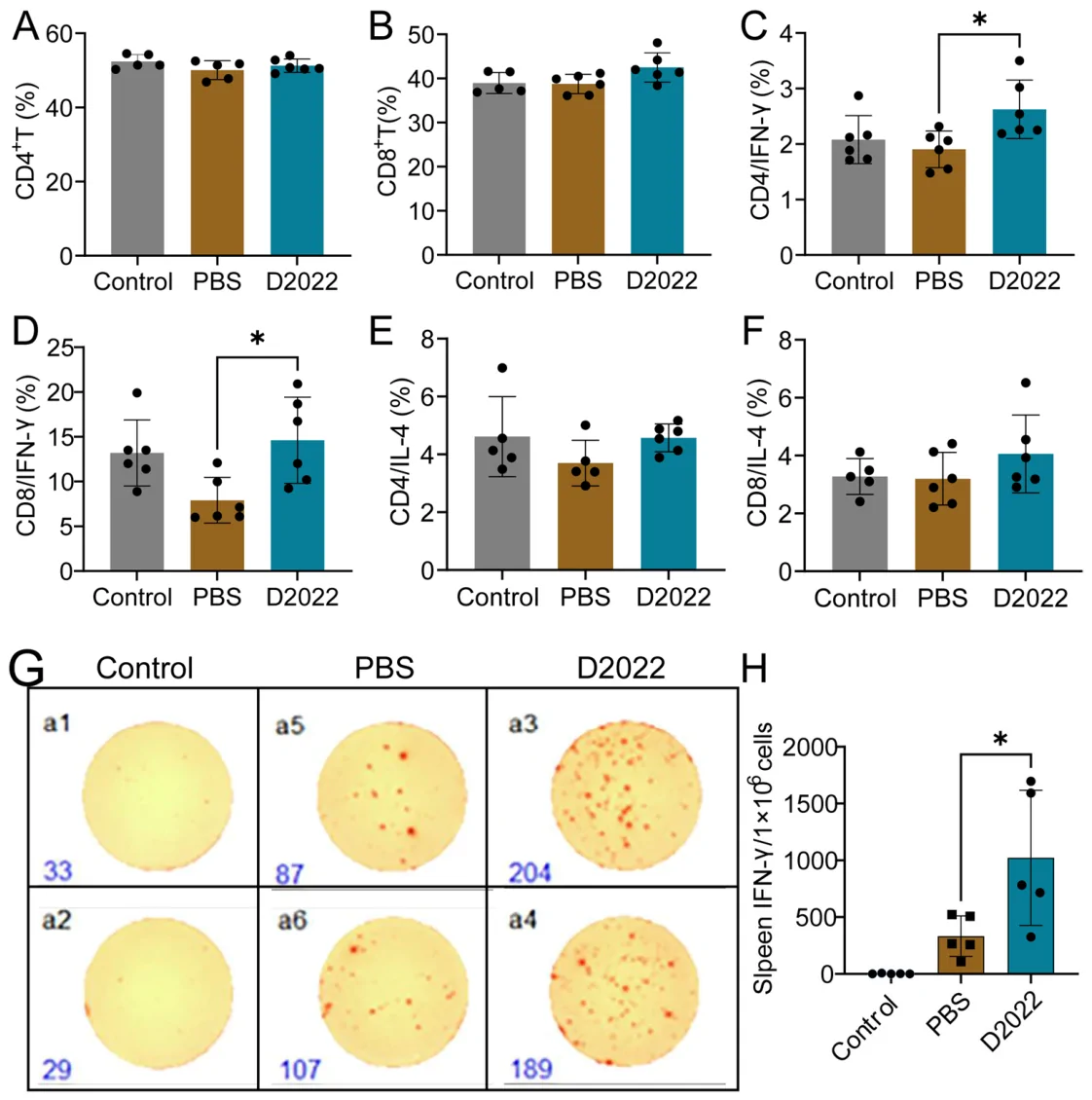
Impact of administration of D2022 on adaptive immune responses against influenza virus in mice. Percentages of CD4^+^ T cells (**A**), CD8^+^ T cells (**B**), CD4^+^ T cells secreting IFN-γ (**C**), CD8^+^ T cells secreting IFN-γ (**D**), CD4^+^ T cells secreting IL-4 (**E**), and CD8^+^ T cells secreting IL-4 (**F**) in splenocytes of mice of different groups on day 7 post-infection measured by flow cytometry. (**G**) Influenza-specific IFN-γ release of splenocytes of mice of different groups measured by ELISpot stimulated with HA peptides. (**H**) ELISpot counts (SFU) of different groups. Data shown in panels (**A**,**F**) were obtained from one of three independent experiments. * *p* < 0.05.

Flow cytometry analysis revealed a significant increase in IFN-γ-secreting CD4^+^ T cells and CD8^+^ T cells in D2022-treated mice compared to PBS-treated mice (Figure 9A–D). Additionally, ELISpot assays showed a higher number of influenza-specific IFN-γ-producing T cells in the D2022-treated mice (Figure 9G,H). These findings suggest that *L. cremoris* D2022 treatment induced an earlier adaptive cellular immune response against the influenza virus compared to PBS treatment.

## 4. Discussion

In this study, we demonstrate that *L. cremoris* D2022, a dominant probiotic isolated from Douzhier—a traditional Chinese fermented food with a history of approximately 1000 years—confers protection against IAV infection in mice, similar to the previously reported *Lactococcus cremoris* FC strain derived from Caspian Sea yogurt [11]. Oral administration of *L. cremoris* D2022 increased the survival rate of IAV-infected mice from 0% to 30%, accompanied by attenuation of inflammatory responses in the lung and colon, reduced body weight loss, and a marked decrease in pulmonary viral load.

Kinetic analysis revealed that *L. cremoris* D2022 treatment significantly reduced viral load in the lungs at 3 days post-infection (dpi), whereas viral titers in both groups declined to comparable levels at later stages. These findings suggest that *L. cremoris* D2022 primarily restricts viral replication during the early phase of infection.

An increasing body of evidence has demonstrated anti-influenza activity across diverse probiotic taxa, including *Bacteroides dorei* [22], *Lactococcus lactis* [11], *Lactobacillus plantarum* [12,13], *Leuconostoc mesenteroides* [14], *Akkermansia muciniphila* [7], and *Bacteroides fragilis* [16]. A central mechanism underlying these effects is the induction of type I interferons (IFNs) by microbial components or metabolites.

Consistent with this paradigm, co-culture experiments showed that *L. cremoris* D2022 robustly induced IFN-β expression in mouse bone marrow-derived dendritic cells (mBMDCs). In vivo, IFN-β expression in the lungs was significantly elevated at 1 dpi in D2022-treated mice compared to PBS controls. Transcriptomic profiling further revealed upregulation of interferon-stimulated genes (ISGs), including *Ifna1*, *Oas*, and *Isg15*, indicating activation of the IFN signaling pathway.

Mechanistically, prior work by Steed et al. [27] demonstrated that *Clostridium orbiscindens* metabolizes dietary flavonoids into desaminotyrosine (DAT), a metabolite that enhances type I IFN responses and protects against influenza infection. Notably, we found that *L. cremoris* D2022 exhibits strong quercetin-degrading activity—a flavonoid structurally analogous to substrates utilized by *C. orbiscindens*—and significantly increases intestinal DAT levels in IAV-infected mice following D2022 treatment. Together, these findings suggest that *L. cremoris* D2022 may promote type I IFN responses through flavonoid metabolism and DAT production, thereby restricting early viral replication. Given that type I IFN signaling represents a broad antiviral mechanism, this effect is unlikely to be influenza-specific and may extend to other respiratory viral infections.

As expected, *L. cremoris* D2022 demonstrated immunomodulatory activity consistent with that of other *L. cremoris* strains, such as FC and YRC3780. At the late stage of infection (7 dpi), levels of multiple pro-inflammatory cytokines—IL-1β, IL-6, TNF-α, and KC—were significantly reduced in the serum and lungs of IAV-infected mice treated with *L. cremoris* D2022 compared to the untreated model group. Lung transcriptome analysis showed significant downregulation of genes involved in the *NF-κB*, *NOD*-like receptor, *IL-17*, and *TNF* signaling pathways. Western blotting further confirmed reduced expression of key proteins in the NF-κB pathway, including phosphorylated NF-κB p65 and IκBα. In contrast, at the early stage of infection (1 dpi), transcriptome comparisons between the D2022 and model groups did not reveal similar alterations in inflammation-related signaling pathways. These results suggest that *L. cremoris* D2022 suppresses the overexpression of pro-inflammatory cytokines and chemokines during the later stages of IAV infection, thereby alleviating inflammation-mediated injury.

To elucidate the immunomodulatory mechanisms of *L. cremoris* D2022, we performed metabolomic profiling of intestinal contents and identified increased levels of short-chain fatty acids (SCFAs) and secondary bile acids. SCFAs, including acetate, propionate, and butyrate, are produced by microbial fermentation of dietary fiber and are key regulators of mucosal immune homeostasis. *L. cremoris* D2022 treatment significantly elevated propionate and butyrate levels in the cecum. SCFAs are important microbiota-derived immunoregulatory metabolites. Previous studies have shown that butyrate and propionate promote the extrathymic differentiation of Foxp3^+^ regulatory T (Treg) cells, at least in part through histone deacetylase inhibition and epigenetic regulation of Foxp3 [28]. Consistent with these findings in murine systems, a human study demonstrated that butyrate and propionate enhance the differentiation and suppressive function of induced Treg cells derived from naïve human CD4^+^ T cells, partly through modulation of histone acetylation [29]. In the present study, D2022 treatment significantly increased cecal propionate and butyrate levels. Therefore, the D2022-associated increase in SCFAs may contribute to the attenuation of excessive inflammation through Treg-related immunoregulatory mechanisms. However, because Treg frequency and Foxp3 expression were not directly measured in the present study, this mechanism remains to be experimentally validated.

Notably, our previous work also showed that D2022 increased intestinal SCFA levels in a murine model of hyperuricemia [21], further supporting its potential capacity to modulate microbial metabolic activity. In contrast, acetate levels were not significantly altered in the present study. This discrepancy may reflect differences in gut microbiota composition, dietary background, or the availability and composition of fermentable prebiotic substrates across experimental models.

The potential influence of dietary prebiotics on microbial colonization and metabolite production is also supported by recent evidence from studies of early-life nutrition. Den Elzen et al. [30] reviewed evidence indicating that human milk components with prebiotic properties, particularly human milk oligosaccharides and polyphenols, can influence the establishment of beneficial gut microorganisms in infants and may contribute to protection against allergic diseases. These observations highlight the close interaction between dietary substrates, intestinal microorganisms, and microbiota-derived metabolites in shaping host immune responses.

Taken together, our findings suggest that the anti-influenza effects of D2022 may be mediated, at least in part, through alterations in microbiota-derived metabolites such as SCFAs. However, this efficacy may depend on the availability and composition of dietary prebiotic substrates.

Future studies incorporating defined prebiotic components, either alone or in combination with D2022, are therefore warranted to determine how diet–microbe interactions influence the immunomodulatory and anti-influenza effects of D2022 and to more accurately characterize the conditions under which its protective efficacy is optimized.

Secondary bile acids, generated through microbial transformation of primary bile acids (e.g., CA and CDCA), have emerged as important immunoregulatory metabolites. Recent studies have identified specific microbial conjugate bile acids that modulate T cell differentiation, e.g., 3-oxoLCA and iso-LCA suppresses Th17 cell responses via inhibition of RORγt, whereas isoalloLCA promotes Treg differentiation through induction of *Foxp3* [31,32]. In addition, CDCA-derived metabolites such as isoLCA can reprogram macrophage metabolism and attenuate inflammatory cytokine production in the lung [33]. Notably, *L. cremoris* D2022 treatment markedly increased the levels of ursodeoxycholic acid (UDCA), isoUDCA and isoalloLCA. UDCA, a clinically used bile acid for primary biliary cholangitis, has also been shown to promote Treg differentiation via NR4A1-dependent upregulation of *Foxp3* as isoalloLCA [33,34]. These findings indicate that, in addition to SCFA-mediated effects, *L. cremoris* D2022 may exert immunomodulatory activity through remodeling of the bile acid pool, thereby enhancing Treg responses. Collectively, D2022 treatment was associated with increased levels of SCFAs and immunoregulatory bile acid metabolites, which may contribute to the attenuation of excessive inflammation.

Beyond innate immunity, *L. cremoris* D2022 enhanced adaptive responses to IAV. At 7 dpi, flow cytometry showed increased frequencies of IFN-γ-producing CD4^+^ and CD8^+^ splenocytes in D2022-treated mice relative to PBS controls. Consistent with the flow cytometry results, ELISpot analysis revealed a robust increase in influenza-specific IFN-γ-secreting cells, indicating enhanced antigen-specific T cell responses. These data support a role for *L. cremoris* D2022 in promoting adaptive cellular immunity. Consistent with this, Saito et al. demonstrated that *L. cremoris* FC augments adaptive immunity by enhancing MHC class I-restricted antigen presentation [10]. Accelerated induction of virus-specific T cell responses by *L. cremoris* D2022 likely contributes to improved viral clearance and reduced disease duration.

Douzhier is a traditional Chinese fermented food derived from mung beans, with a history spanning over 1000 years. Similar to two well-known fermented dairy products—Caspian Sea yogurt and kefir, both originating from the Caucasus region and consumed for more than 700 years—these long-standing fermented foods harbor complex microbial ecosystems in which *Lactococcus cremoris* is a predominant species. Previous studies have shown that *L. cremoris* strains isolated from these dairy products can modulate host immunity, including suppression of allergic inflammation and protection against viral infection. In this study, we identify *L. cremoris* D2022 as a predominant microbial component of *Douzhier* and demonstrate its capacity to enhance host responses to IAV infection. Integrated with our mechanistic data, these findings suggest that *L. cremoris* D2022 may modulate host–microbiota interactions to support both innate and adaptive immunity against influenza. *L. cremoris* accounts for 38–55% of the Douzhier microbiota, we observed total bacterial loads of 3 × 10^9^ CFU/100 mL, corresponding to an estimated *L. cremoris* D2022 abundance of 1.14–1.65 × 10^9^ CFU/100 mL—within the range reported for functional probiotic foods. Given its long history of dietary use, low cost, and accessibility, *Douzhier* represents a potentially practical adjunct for supporting antiviral immunity at the population level.

Several limitations of the present study should be acknowledged. First, the current experiments evaluated the isolated *Lactococcus cremoris* D2022 strain rather than Douzhier itself. Because Douzhier is a complex fermented food containing diverse microorganisms, soluble amino acids, and other fermentation-derived metabolites, the effects observed for D2022 as a single isolated probiotic strain cannot be directly extrapolated to those of the whole fermented product. Second, although D2022 administration was associated with changes in intestinal DAT, SCFAs, and bile acid metabolites, these findings do not establish a causal role for these metabolites in mediating the observed protection. Further mechanistic studies involving metabolite supplementation or depletion, receptor blockade, and microbiota manipulation will be required to determine whether these metabolic changes directly contribute to the protective effects of D2022. Third, an oseltamivir-treated positive-control group was not included because the primary aim of this study was to investigate the mechanisms underlying D2022-mediated protection rather than to directly compare its antiviral efficacy with that of a standard anti-influenza drug. Future comparative studies incorporating oseltamivir or other established antiviral agents will be valuable for more comprehensively evaluating the anti-influenza efficacy of D2022. Finally, the present findings were obtained in a murine model and have not yet been validated in humans. Further clinical studies are therefore needed to evaluate the safety, efficacy, reproducibility, and durability of D2022-mediated protection and to determine whether probiotic-based interventions could complement existing influenza prevention strategies. Collectively, these limitations define important directions for future investigation while providing a framework for further validation and mechanistic characterization of the present findings.

## 5. Conclusions

In conclusion, our study demonstrated that *L. cremoris* D2022 provides protective efficacy against IAV infection in mice. The underlying mechanisms likely involve inhibiting viral replication through increasing IFN-β levels rapidly, probably with metabolite (DAT) in the early stages of infection, and reducing excessive inflammatory responses, probably with SCFAs and microbial conjugate bile acids in the late stages. These observations support a potential metabolite-associated mechanism, although direct causal relationships remain to be established. These findings suggest that *L. cremoris* D2022, and even Douzhier, hold promise as auxiliary prophylactic measures for IAV infection.

## Data Availability

The data that support the findings of this study are available from the corresponding author upon reasonable request. Additionally, The Transcriptome original data were deposited in the NCBI SRA database (accession number: PRJNA1100256 for day 1; PRJNA1102099 for day 7). The genome sequencing data of L. cremoris D2022 strain were deposited in the NCBI Genbank (BioProject No. PRJNA1014919).

## Supplementary Materials

The following supporting information can be downloaded at https://www.mdpi.com/article/10.3390/nu18183089/s1, Figure S1. Comparative genomic analysis of *Lactococcus cremoris* D2022 and representative *L. cremoris* strains. Table S1. Genomic characteristics of *Lactococcus cremoris* D2022 and representative *L. cremoris* strains. Genome size, GC content, number of assembly sequences, and sequence N50 were obtained from the corresponding NCBI RefSeq assemblies. Coding sequences (CDSs), rRNA genes, and tRNA genes were consistently predicted for all strains using Prokka v1.14.5. Average nucleotide identity (ANI) relative to D2022 was calculated using FastANI. Table S2. Comparative analysis of selected functional genes associated with bile acid metabolism in Lactococcus cremoris D2022 and representative *L. cremoris* strains. Experimentally characterized or well-annotated proteins were selected as reference sequences for homology searches. Reference proteins were searched against the Prokka-predicted proteomes using DIAMOND BLASTP. Values indicate amino acid sequence identity (%)/query coverage (%) of the best-scoring hit. A dash (–) indicates that no significant homolog satisfying the predefined search criteria (E-value ≤ 1 × 10^−5^ and query coverage ≥ 50%) was detected. † BSH-related hits exhibited extensive query coverage but relatively low amino acid sequence identity and were therefore conservatively classified as candidate BSH-family homologs pending further verification of conserved domains and catalytic residues. Figure S2. Assay for 24-hour quercetin degradation.
