# Supplementary material for: A Dominant Probiotic from the Traditional Chinese Fermented Food Douzhier Exhibits Anti-Influenza Activity
Source: Nutrients. 2026 Sep 21;18(18):3089. doi: 10.3390/nu18183089 (PMC13610886; doi:10.3390/nu18183089)
Supplement: Supplementary file 1 [file nutrients-18-03089-s001.zip › nutrients-4519386-supplementary.pdf]

Supplementary Materials

Figure S1. Comparative genomic analysis of *Lactococcus cremoris* D2022 and representative *L. cremoris* strains.

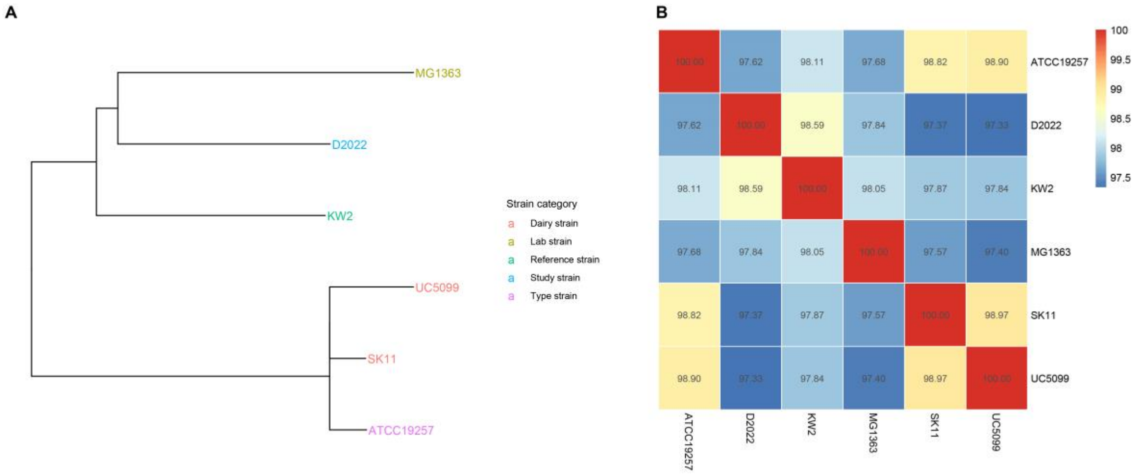

(A) Phylogenomic tree of *Lactococcus cremoris* D2022 and representative *L. cremoris* strains constructed from core-genome alignments using Parsnp. (B) Pairwise average nucleotide identity (ANI) heatmap of the same strains calculated using FastANI. ANI values are expressed as percentages.

Table S1. Genomic characteristics of *Lactococcus cremoris* D2022 and representative *L. cremoris* strains. Genome size, GC content, number of assembly sequences, and sequence N50 were obtained from the corresponding NCBI RefSeq assemblies. Coding sequences (CDSs), rRNA genes, and tRNA genes were consistently predicted for all strains using Prokka v1.14.5. Average nucleotide identity (ANI) relative to D2022 was calculated using FastANI.

**Strain**	**Assembly accession**	**Genome size (Mb)**	**GC content (%)**	**No. of sequences**	**Sequence N50 (kb)**	**CDS**	**rRNA**	**tRNA**	**ANI with D2022 (%)**
D2022	GCF_031583695.1	2.63	35.42	65	132.5	2610	6	53	100.00
ATCC19257	GCF_004354515.1	2.57	36.02	213	20.2	2596	28	69	97.62
MG1363	GCF_000009425.1	2.53	35.75	1	2529.4	2580	19	62	97.84
KW2	GCF_000468955.1	2.43	35.74	1	2427.0	2285	19	61	98.59
SK11	GCF_000014545.1	2.60	35.78	6	2438.6	2714	20	62	97.37
UC5099	GCF_000312685.1	2.46	35.74	9	2250.4	2534	19	60	97.33

Table S2. Comparative analysis of selected functional genes associated with bile acid metabolism in Lactococcus cremoris D2022 and representative *L. cremoris* strains. Experimentally characterized or well-annotated proteins were selected as reference sequences for homology searches. Reference proteins were searched against the Prokka-predicted proteomes using DIAMOND BLASTP. Values indicate amino acid sequence identity (%) / query coverage (%) of the best-scoring hit. A dash (–) indicates that no significant homolog satisfying the predefined search criteria (E-value $\leq 1 \times 10^{-5}$ and query coverage ≥ 50%) was detected. †BSH-related hits exhibited extensive query coverage but relatively low amino acid sequence identity and were therefore conservatively classified as candidate BSH-family homologs pending further verification of conserved domains and catalytic residues.

**Functional category**	**Gene**	**Reference accession**	**D2022**	**ATCC19257**	**MG1363**	**KW2**	**SK11**	**UC5099**
Bile acid metabolism	bsh	A0A805Z5R7	24.8 / 96.8†	–	–	25.2 / 96.8†	25.3 / 75.6†	25.3 / 75.6†
Bile acid metabolism	baiE	WP_006443993.1	–	–	–	–	–	–

Figure S2

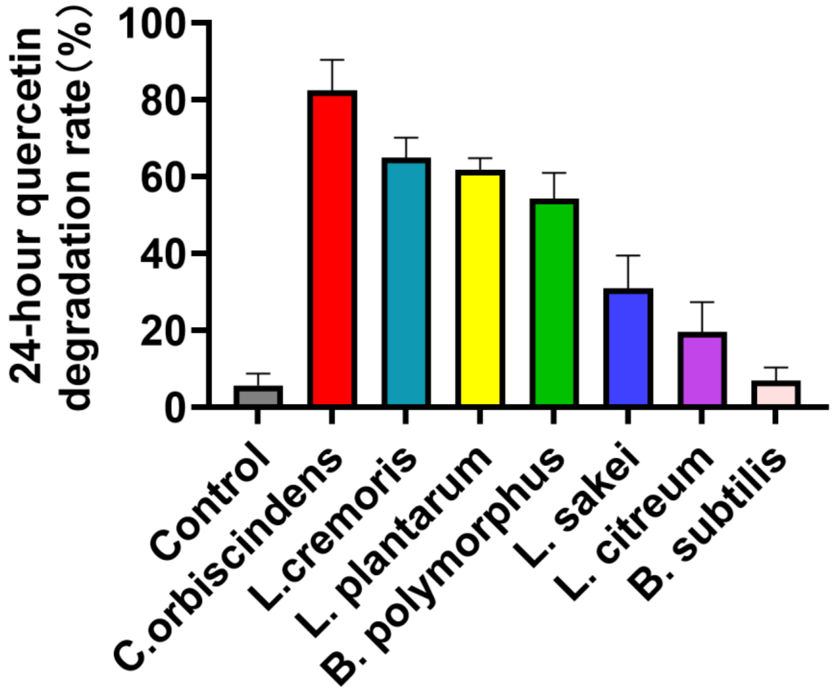

Figure S2. Assay for 24-hour quercetin degradation

Comparison of seven probiotics for quercetin degradation efficacy: Samples 1–8 from left to right are: Negative control group (Control, 5.6%), Positive control group (Clostridium orbiscindens, C. orbiscindens , 82.3%), Lactococcus cremoris group (L. Cremoris D2022, 65%), Lactobacillus plantarum group (L. plantarum 61.7%); Bacteroides polymorphus group (B. polymorphus, 54.2%), Lactobacillus sakei group (L. sakei, 30.9%), Leuconostoc citreum group (L. citreum, 24.3%), Bacillus subtilis group (B.Subtilis, 6.8%), Nos. 6–8 showed poor quercetin-degrading activity. The data in panel are

means ± SD of three independent experiments.
